# Psychometric Validation of the Bangla Version of the Breast Cancer Fear Scale Among Female University Students in Bangladesh

**DOI:** 10.1155/ijbc/6811105

**Published:** 2025-04-22

**Authors:** Md. Ashfikur Rahman, Md Mikail Hossen, Md. Ehsanul Haque Chowdhury, Farzana Afrin Anu, Tanjirul Islam, Md. Sazedur Rahman, Satyajit Kundu, Md. Hasan Howlader

**Affiliations:** ^1^Department of Applied Social Sciences, Faculty of Health and Social Sciences, The Hong Kong Polytechnic University, Hong Kong, China; ^2^Development Studies Discipline, Social Science School, Khulna University, Khulna, Bangladesh; ^3^Mass Communication and Journalism Discipline, Social Science School, Khulna University, Khulna, Bangladesh; ^4^Applied Statistics, Institute of Statistical Research and Training (ISRT), Dhaka University, Dhaka, Bangladesh; ^5^Faculty of Social Sciences, Social Welfare, Islamic University, Kushtia, Bangladesh; ^6^Statistics Discipline, Khulna University, Khulna, Bangladesh; ^7^Public Health, School of Medicine and Dentistry, Griffith University, Gold Coast, Queensland, Australia

**Keywords:** Bangla, Bangladesh, breast cancer, fear, psychometric, women

## Abstract

**Background:** Research suggests that fear of cancer could be a significant predictor influencing participation in cancer screening. However, no tools have been validated to measure breast cancer fear among women in Bangladesh, while the Breast Cancer Fear Scale (BCFS) has been extensively examined in Western contexts. Thus, this study intends to validate the Bangla version of the BCFS among female university students aged (> 18) years, given the urgent need for a culturally relevant tool to evaluate fear associated with breast cancer screening practices in this population.

**Methods:** This cross-sectional study was conducted in 2023 among female university students in Bangladesh. Participants were aged > 18 years, able to read Bangla, and had no personal or familial history of cancer or chronic illnesses. Data were collected via an online survey using a random sampling method, resulting in 456 eligible participants after data cleaning. The BCFS was translated into Bangla following the standard forward–backward translation process. Exploratory and confirmatory factor analyses (EFA and CFA) were conducted to evaluate the structure of the scale factor. Internal consistency, test–retest reliability, and convergent validity were also assessed.

**Results:** The results showed that the mean age of the participants was 22.91 (SD: 1.12). The Bangla version of the BCFS showed a single-factor structure, high internal consistency (Cronbach's *α* = 0.939), and good test–retest reliability (*r* = 0.53, *p* < 0.001). The CFA results are consistent with the EFA findings, confirming that the scale is a good fit for the one-factor structure. The loadings range from 0.679 (Fear1) to 0.920 (Fear4) in the total sample, indicating that the items are significant indicators of the latent construct. The BCFS demonstrated an acceptable model fit, with RMSEA values below the 0.08 cutoff and SRMR values well below the 0.05 threshold across all samples. Additionally, the GFI, AGFI, NFI, TLI, and CFI values were all above the recommended thresholds, indicating a high fit for the model.

**Conclusions:** The Bangla version of the BCFS has proven to be a powerful and reliable tool for gauging the multifaceted nature of breast cancer fear among Bangladeshi women, particularly female university students. This culturally tailored instrument holds the potential to shed light on the psychological barriers that hinder breast cancer screening.

## 1. Introduction

Breast cancer is a significant public health issue globally and is the most common cancer affecting women. Breast cancer is a category of cancer that develops in the cells of the breast and initially appears as a lump, either alone or in combination with other symptoms [1]. It is among the most prevalent progressive malignancies in women worldwide and the second most common cancer among women [2, 3] while breast cancer is the most frequently diagnosed female cancer and the leading cause of cancer death in women worldwide [4]. In 2022, 2.3 million women were diagnosed with breast cancer, resulting in 670,000 fatalities worldwide, breast cancer occurs at any age postpuberty, with prevalence escalating in later years [5]. Out of 185 countries, breast cancer was the most prevalent form of cancer in females in 157 of those countries in 2022 [5]. However, worldwide approximations demonstrate notable disparities in the incidence of breast cancer based on human development index (HDI) [5]. For example, one in 12 women may receive a breast cancer diagnosis in their lifetime, and one in 71 will pass away from the disease in nations with a very high HDI while only one in 27 women in low-HDI nations receive a breast cancer diagnosis during their lifetime; nonetheless, one in 48 of them pass away from the disease [5].

Developed and underdeveloped countries have different incidence and fatality rates from breast cancer. Australia/New Zealand, Northern Europe (e.g., the United Kingdom, Sweden, Finland, and Denmark), Western Europe (Belgium [with the highest global rates], the Netherlands, and France), Southern Europe (Italy), and Northern America have the greatest incidence rates of breast cancer [6]. Almost half of the incidence (45.4%) and mortality (50.6%) cases were observed in Asia [7]. Asian countries were responsible for about half of the cases, both in terms of incidence (45.4%) and death (50.6%). In the United States, invasive breast cancer affects one in 8 women (13%) at some point in their lives. Breast cancer is the most common cancer among women, accounting for 30% of all new cases diagnosed each year, with two-thirds being classified as localized diseases [8, 9].

In Bangladesh, where health literacy is often limited and cultural stigma surrounds discussions of cancer, fear of breast cancer can become a considerable barrier to early diagnosis and treatment [10]. Fear is a psychological response that can lead to avoidance behaviors, such as reluctance to engage in screening programs or seek medical help, potentially resulting in delayed diagnosis [11]. The Breast Cancer Fear Scale (BCFS), developed by Champion et al. [12], is a widely used instrument designed to assess fear related to breast cancer. However, its applicability to non-Western contexts, such as Bangladesh, where cultural, linguistic, and social factors significantly shape health perceptions, necessitates psychometric validation of a localized version.

One study conducted in South Asian countries discovered that there was a total of 200,000 cases of breast cancer, of which 97,500 people passed away in the year 2012 [13]. Among the leading causes of death in Bangladesh, breast cancer is one of the prominent causes among women [14]. In Bangladesh, breast cancer is the leading cause of mortality among women, accounting for 18.8% of all cancer cases and 7.8% of all malignancies, in 2022 [15]. Breast cancer mortality rates in Bangladesh are greater due to a lack of self-awareness, understanding and knowledge, limited access to proper treatment and diagnostic facilities, and community negligence [16]. Even though it is a burden on society, people are talking less about this illness while lack of awareness and education are major reasons why breast self-examination (BSE) is not practiced in Bangladesh [17]. Inadequate information on causes and risk factors, communities' lack of knowledge about self-diagnosis and breast cancer therapy delays treatment, and unfriendly social environments hinder women from discussing breast complaints or changes with others [16]. Because of this, advanced-stage presentations in Bangladesh are frequently delayed [18], resulting in 50% of breast cancer patients having died due to late presentation and in an advanced stage [19].

The psychometric validation of a translated instrument ensures its reliability and validity within the target population, allowing researchers and healthcare professionals to assess the psychological construct it intends to measure accurately [20]. In Bangladesh, a country with a growing incidence of breast cancer but limited mental health services, there is an urgent need for a culturally adapted version of the BCFS that can accurately capture the fear experienced by women. Fear of breast cancer can influence health-seeking behaviors, participation in screening programs, and overall mental health, highlighting the importance of a valid and reliable scale to measure this construct [21]. The burden of the disease can be significantly reduced by early identification and treatment; nevertheless, screening and early diagnosis have been found to be impeded by breast cancer [22]. This fear frequently results in avoiding mammograms and other preventive actions, which raises the possibility of a late-stage diagnosis and unfavorable results [23, 24]. The Champion's BCFS has been validated in multiple cultural contexts and is extensively used to measure fear associated with breast cancer [25, 26].

This study aims to assess the psychometric properties (including reliability, validity, and factor structure) of the Bangla version of the BCFS among female university students in Bangladesh. The study seeks to evaluate the scale's internal consistency, construct validity, and applicability within this population to ensure its effectiveness in measuring breast cancer fear in the Bangladeshi context. This validation will provide a tool that can be used in both clinical and research settings to understand better the role of fear in breast cancer prevention and treatment. Psychometric properties, including reliability, factor structure, and construct validity, will be examined to ensure the scale's appropriateness in this population. By providing a validated scale, this study will contribute to a better understanding of how fear influences breast cancer-related behaviors and may help in designing interventions to reduce fear and encourage early detection and treatment in Bangladesh.

## 2. Methods

### 2.1. Study Design and Participants

In this study, the cross-sectional survey design was followed to collect the data through online questionnaire to evaluate the psychometric properties of the Bangla version of the Champion's BCFS among female university students in Bangladesh who met the following criteria: (a) aged 18 years or older, (b) able to read Bangla, (c) with no personal or familial history of breast cancer, (d) with no personal or familial history of any other type of cancer, (e) not currently diagnosed with a mental illness or receiving medication for such conditions, and (f) without any prior chronic illnesses (e.g., heart disease, history of stroke, respiratory diseases, and chronic obstructive pulmonary disease (COPD). The sample size was determined based on the guideline of having 5 to 10 participants per item on the scale [27, 28]. Initially, data was collected from 500 participants, randomly sending them online questionnaires through WhatsApp, Messenger, and Email. However, after data cleaning and inconsistency checks, 44 cases were removed, leaving 456 eligible samples for final analysis. The dataset was randomly split into two groups for both exploratory factor analysis (EFA) and confirmatory factor analysis (CFA). Both EFA and CFA were conducted on the overall dataset as well. The final sample of 456 participants, which exceeds the minimum requirement of 200, is sufficient to ensure reliable results in CFA [29].

### 2.2. Translation Process

The BCFS is an 8-item self-report instrument in English Language designed to evaluate fear associated with breast cancer [12], created and validated the BCFS to assess breast cancer fear in women. The BCFS was translated into Bangla following the standard forward–backward translation method recommended by Beaton et al. [20]. First, two independent bilingual translators (two graduates from the English department), fluent in both English and Bangla, independently translated the original English version into Bangla. A third expert (PhD student carrying out PhD in Psychology) proficient in both English and Bangla synthesized the translations into a single version. This version was then meticulously back-translated into English by two independent translators who had yet to see the original scale, ensuring a thorough and accurate process. The back-translated version was compared to the original BCFS to ensure semantic equivalence. Any discrepancies were resolved through discussion. The final Bangla version of the BCFS was pretested on 20 women to ensure clarity and cultural appropriateness. Minor modifications were made based on participant feedback.

### 2.3. Measures

The Bangla-translated version of BCFS was used to assess the fear of breast cancer among female university students in Bangladesh. It aims to capture the unidimensional of this construct, specifically assessing fear of diagnosis by inquiring about concerns regarding the potential of being diagnosed with breast cancer. The BCFS utilizes a 5-point Likert scale, with responses ranging from 1 (*significantly disagree*) to 5 (*significantly agree*). This format enables respondents to express varying degrees of agreement or disagreement with each statement, thereby providing nuanced data on their levels of fear. Higher total scores on the BCFS suggest a greater fear of breast cancer, offering valuable insights for healthcare professionals into patients' psychological barriers to seeking care or engaging in preventive measures [12]. The scale demonstrated significant reliability and validity, with a Cronbach's alpha of 0.91 and a test–retest correlation of 0.70 in previous research [12]. We also collect the socio-demographic information of the study participants.

### 2.4. Statistical Analysis

To assess the psychometric properties of the Bangla version of the BCFS, several statistical analyses were conducted. They are described in the following:

▪ *Reliability:* Internal consistency was evaluated using Cronbach's alpha. A value of 0.70 or higher was considered acceptable [30].
▪ *Test–retest reliability*: A subsample of 20 participants completed the BCFS again after 2 weeks to assess test–retest reliability. Intraclass correlation coefficients (ICCs) were calculated.
▪ *Construct validity:* EFA with principal component analysis (PCA) was performed to explore the underlying factor structure of the BCFS. Bartlett's test of sphericity and the Kaiser–Meyer–Olkin (KMO) measure were used to assess the suitability of the data for factor analysis, where Promax rotation was used since underlying constructs were expected to be correlated.

Finally, the factor structure of the BCFS was evaluated using CFA. The covariance matrix of the associated items for the scale was fitted with a one-factor model that included all the items as indicators. Using SPSS, we randomly divided the sample in half to conduct a crossvalidation analysis of the model to assess the stability of the factor structure of the scale. All the CFAs were performed using SmartPLS 4.1.0.9 version while other analyses were performed using IBM SPSS.25 and Jamovi 2.2.8 version. In all statistical analyses, a *p* value of less than 0.05 is deemed statistically significant. CFA was conducted to assess the factor structure of the BCFS. A one-factor model with all the items as indicators was fitted to the covariance matrix of the corresponding items for the scale. To test the stability of the factor structure of the scale, we performed a crossvalidation examination of the model by randomly splitting the sample into half using SPSS. Three fit indices—the Robust Comparative Fit Index (R-CFI) [31]; the Standardized Root Mean Squared Residues (SRMR) [32]; and the Root Mean Square Error of Approximation (RMSEA); [33]—were used to evaluate how well the model fits the data. A model that fits the data well and will not be rejected has values of R − CFI > 0.90, SRMR < 0.08, and RMSEA < 0.08 [34]. For model verification, further multigroup invariance (MICOM and MGA), inter-item correlation, and Cronbach alpha, along with McDonald's Omega internal consistency measurement, were tested.

### 2.5. Ethical Considerations

The study received ethical approval from the Khulna University Ethical Clearance Committee (KUECC-2024-10-74). We thoroughly informed all participants about the study's purpose, the voluntary nature of their participation, and the confidentiality of their responses. Prior to collecting data, we obtained informed consent from all participants. No identifiable information was collected to maintain participants' anonymity. Furthermore, we stored all data securely with strict confidentiality measures to protect participants' privacy and confidentiality.

## 3. Results

### 3.1. Background Characteristics of the Study Samples

Table 1 presents the demographic and socioeconomic characteristics of the study participants, divided into two subsamples, Split Sample 1 (*N* = 224) And Split Sample 2 (*N* = 232), as well as the total sample (*N* = 456). The age of participants in both groups is very similar, with a slightly higher mean in Split Sample 1. The standard deviation is larger in Split Sample 2, indicating more variation in the ages of participants. Both groups are predominantly unmarried, with Split Sample 2 having a slightly higher percentage of unmarried participants. The difference between the two groups is minimal. The income distribution is similar between the two groups, with Split Sample 2 having a slightly higher percentage of participants in the lowest income group (< 10,000 BDT) and the middle-income group (10,000–30,000 BDT). Split Sample 1, on the other hand, has more participants in the highest income group (> 50,001 BDT) than Split Sample 2, where this group is less represented. Split Sample 2 has more smokers (8.0%) compared to Split Sample 1 (3.4%), indicating a notable difference in smoking history between the groups. A higher proportion of participants in Split Sample 1 are from urban areas compared to Split Sample 2. Conversely, Split Sample 2 has more participants from rural areas.

### 3.2. Factor Structure and Reliability

Table 2 presents the results of the EFA and CFA for the BCFS, along with the Cronbach's alpha for internal consistency reliability. The data are analyzed for the overall sample and the two split samples, providing insights into the scale's psychometric properties. The mean scores for individual items on the BCFS range from 3.59 to 4.09 across all participants, indicating a generally high level of fear related to breast cancer. The relatively small standard deviations suggest that participants responded somewhat consistently to the items.

The factor loadings in the EFA for the total sample range from 0.733 (Fear1) to 0.904 (Fear4), indicating that all items load significantly onto the single factor. Typically, loadings above 0.70 are considered good, showing that the items are well correlated with the underlying construct (breast cancer fear). In the split samples: Split Sample 1 shows factor loadings ranging from 0.602 (Fear1) to 0.899 (Fear4) while Split Sample 2 has factor loadings ranging from 0.772 (Fear7) to 0.908 (Fear4). In both subsamples, the factor loadings are similarly significant, with some variation across items, particularly for Fear1 (lower in Split Sample 1) and Fear7 (lower in Split Sample 2).

The CFA results are generally consistent with the EFA findings, confirming that the scale is a good fit for the one-factor structure. The loadings range from 0.679 (Fear1) to 0.920 (Fear4) in the total sample, indicating that the items are significant indicators of the latent construct. In the split samples: Split Sample 1 CFA loadings range from 0.539 (Fear1) to 0.921 (Fear4) while Split Sample 2 CFA loadings range from 0.711 (Fear7) to 0.914 (Fear4). There is a noticeable difference in the factor loading for Fear1 in Split Sample 1 (0.539) compared to the total sample and Split Sample 2, where it is higher. This suggests some variability in how significantly this item measures fear of breast cancer in the subsamples. The KMO values are high across the total sample (0.934), Split Sample 1 (0.918), and Split Sample 2 (0.937), indicating that the sampling is adequate for factor analysis. Values above 0.90 are considered high, demonstrating that the correlations between items are sufficiently significant to perform factor analysis. In addition, the Cronbach's alpha for the overall sample is 0.939, indicating high internal consistency reliability. This suggests that the items on the BCFS measure the same underlying construct (breast cancer fear) consistently. In split samples: Split Sample 1 has a Cronbach's alpha of 0.933, indicating similarly high internal consistency while in the Split Sample 2 shows a slightly higher Cronbach's alpha of 0.945, suggesting that the scale performs well across both groups with very high reliability, while the McDonald's test shows an acceptable range of internal consistency.

The BCFS shows high psychometric properties in terms of factor structure and reliability. The scale performs well overall and in both split samples, though there are some minor variations in how certain items, particularly Fear1 and Fear7, load onto the underlying construct. These results suggest that the scale is a valid and reliable tool for measuring the fear of breast cancer among women in Bangladesh. However, the variability in factor loadings for certain items between split samples suggests that further investigation may be warranted to explore whether these items behave differently in subgroups of the population.

### 3.3. Model Fit Indexes for the CFA

The RMSEA in (Table 3) values for all samples are below the cutoff of (RMSEA ≤ 0.008), indicating an acceptable model fit. Split Sample 1 shows a slightly better fit (RMSEA = 0.064) compared to the total sample (RMSEA = 0.071) and Split Sample 2 (RMSEA = 0.075), but all remain within the acceptable range. All SRMR values are well below the (SRMR = ≤0.05) threshold, indicating a high fit of the model in all samples. The SRMR values are very close to the total and split samples, with Split Sample 1 having a slightly higher (SRMR = 0.026) but still within the acceptable range. All the Goodness-of-Fit Index (GFI) values are well above the recommended threshold of (GFI = ≥0.9), suggesting a good fit of the model in all samples. The total sample has the best (GFI = 0.974), though the split samples also indicate significant model fit (GFI = 0.969 and 0.966). The Adjusted Goodness of Fit Index (AGFI) values for all samples are above (≥ 0.9), indicating an acceptable model fit. While Split Sample 2 has a slightly lower AGFI (0.918), it is still above the standard threshold. The Normed Fit Index (NFI) values across all samples are very high (all above 0.978), indicating a high model fit. The total sample has the highest NFI (0.983), but the split samples also demonstrate a high fit. The Tucker–Lewis Index (TLI) values are well above 0.9, indicating a high model fit. Split Sample 1 (0.982) has the highest TLI, slightly outperforming the total sample and Split Sample 2. All Comparative Fit Index (CFI) values are very high (above 0.988), confirming a high fit for all samples. Split Sample 1 has the best CFI (0.990), but the differences across samples are minimal. The Parsimony Goodness of Fit Index (PGFI) values for all samples are below the desired threshold of 0.5, indicating that the model may not be parsimonious. However, PGFI is generally considered less important than other fit indices, especially when other indicators suggest a significant model fit. Composite reliability is high for all samples, with values well above 0.7. Split Sample 2 shows the highest reliability (0.933), while Split Sample 1 has the lowest but acceptable value (0.905). The Average Variance Extracted (AVE) values for all samples are above the threshold of (AVE ≥ 0.5), indicating that the latent factor explains sufficient variance in the data. Split Sample 2 has the highest AVE (0.678), meaning that the factor explains more variance in this sample than the others.

Tables S1, S2, and S3 contain the detailed results of the inter-item correlation results. The correlation matrix indicates significant and statistically significant positive relationships among all fear items (*p* < 0.001), with inter-item correlations ranging from moderate (*r* ≈ 0.48) to significant (*r* ≈ 0.90). Each item also shows a significant correlation with the total score (*r* = 0.736 to 0.901), suggesting high internal consistency and that all items contribute meaningfully to the overall construct of breast cancer fear. The most significant inter-item correlation is between Fear3 and Fear4 (*r* = 0.838), while Fear4 has the highest correlation with the total score (*r* = 0.901), indicating its significant role in defining the construct. These results support the scale's reliability and justify the use of Promax rotation in factor analysis, as the high correlations suggest the presence of correlated underlying factors rather than completely independent constructs.

While the multigroup invariance (MICOM, MGA) permutation analyses (see Supporting information) revealed that all three reliability measures (rho_c, rho_a, and Cronbach's alpha) show very high values, indicating significant internal consistency across both groups. The differences between the two groups are very small and statistically insignificant, suggesting that the reliability of the scale is consistent across both “residence_1” and “residence_0” groups. In addition, all the permutations (*p* values) are greater than the standard (*p* > 0.05), which establishes that our model can accurately describe its application among the population group.

**Figure 1:**
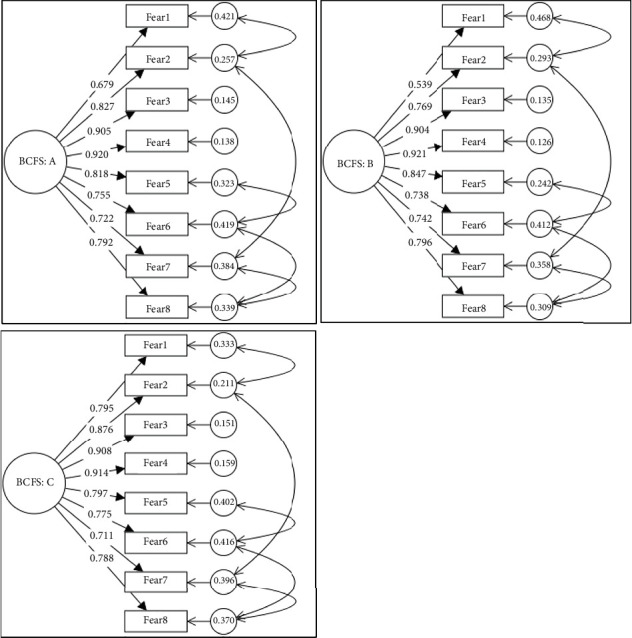
Measurement model for Breast Cancer Fear Scale.

Figure 1 provides a comprehensive view of the relationships between the latent variable (BCFS) and the observed variables (Fear1 to Fear8) in three different models. By analyzing the path coefficients and error terms, we can gain insights into the strength of these relationships and the overall fit of each model. The latent variable (BCFS) has a significant influence on the observed variables (Fear1 to Fear8) in all three models, but the strength of this influence varies. The error terms indicate that there is some unexplained variance in the observed variables, suggesting that other factors may also be influencing these variables. Lower error terms indicate that the latent variable explains a larger portion of the variance in the observed variable. The path coefficients indicate the strength of the relationship between the latent variable (BCFS) and each observed variable (Fear1 to Fear8). Higher coefficients suggest a significant influence of the latent variable on the observed variable. In BCFS: A (BCFS: A, means total sample *N* = 456), the coefficients range from 0.679 to 0.920, indicating varying degrees of influence, while in BCFS: B (BCFS: B, means split 1 sample *N* = 224), the coefficients range from 0.539 to 0.921, with some coefficients being lower compared to BCFS: A. In BCFS: C (BCFS: C, means split 2 sample *N* = 232), the coefficients range from 0.711 to 0.914, generally higher than those in BCFS: B. By comparing the path coefficients and error terms across the three models, we can assess which model provides a better fit.

## 4. Discussion

As far as we are aware, this is the first instrument designed to measure the fear of breast cancer in Bangladeshi women. The present study aimed to validate the Bangla version of the BCFS for use among women in Bangladesh, assessing its psychometric properties. This validation is crucial, as fear of breast cancer can significantly influence health behaviors, such as screening and early detection practices. Given that breast cancer remains a leading cause of morbidity and mortality among women in Bangladesh, having a culturally and linguistically appropriate tool to measure breast cancer fear is essential for both research and clinical practice. The results of this study show that the Bangla version of BCFS has good psychometric qualities, which makes it a valid and acceptable instrument for use in similar settings. The high item–total correlations with the overall score and the significant link with the latent factor show that every item performed well in the studies; the high internal consistency and test–retest reliability of the scale also point to good performance.

The results of the study demonstrated significant evidence for the factorial validity of the Bangla version of the BCFS. A one-factor solution was confirmed, which aligns with the original scale's structure. This suggests that the scale reliably measures a single underlying construct: fear related to breast cancer. Additionally, the scale exhibited high internal consistency, as indicated by a satisfactory Cronbach's alpha and McDonald's value. This level of reliability confirms that the items within the scale are cohesive and measure the construct consistently across different respondents. The concurrent construct validity of the scale was also supported, as the scale correlated well with related constructs, such as anxiety and health-related concerns. These correlations suggest that the scale effectively measures breast cancer fear in relation to broader psychosocial factors, which can provide a more comprehensive understanding of the emotional and psychological aspects tied to breast cancer fear in Bangladeshi women.

This study was designed to take into account eight different categories of fearful thoughts, including (Fear 1: The thought of breast cancer scares me; Fear 2: When I think about breast cancer, I feel nervous; Fear 3: When I think about breast cancer, I get upset; Fear 4: When I think about breast cancer, I get depressed; Fear 5: When I think about breast cancer, I get jittery; Fear 6: When I think about breast cancer, my heart beats faster; Fear 7: When I think about breast cancer, I feel uneasy; and Fear 8: When I think about breast cancer, I feel anxious). The one-factor model is a high fit for data, according to the EFA and CFA carried out in this study. This suggests that all the BCFS's items accurately measure the construction of breast cancer fear as intended. This is in line with the BCFS's initial English version that Champion, and his colleagues created [12]. We are aware of the high prevalence of breast cancer not only in Bangladesh but also globally, which is why the study has chosen this issue. A previous study also discussed this topic in a study they conducted, which showed that breast cancer has surpassed lungs, stomach, colon, and liver cancers as the leading causes of cancer-related deaths globally in 2020 [35]. Furthermore, the severity of breast cancer is particularly severe in developing nations like Bangladesh, and the study has focused on the cultural background of these nations, which is consistent with another study in which the authors make the same specific remark [36]. Additionally, the current study is consistent with a prior investigation that looked at the psychometric qualities of the Depression Anxiety Stress Scales (DASS-21) among breast cancer patients [37]. While another study looked at developing an instrument for the assessment of breast cancer awareness in Thai women [38], this study innovated a tool for the assessment of breast cancer fear among women in Bangladesh.

There is a study on the assessment of stressors in breast cancer patients, as fear and stress both reduce the probability of early breast cancer screening [39]. These findings suggest that the Bangla version of BCFS can be used with confidence to measure fear in Bangladeshi women. These findings are consistent with some earlier research that showed fear, anxiety, and depression are among the psychological barriers that prevent women from participating in early breast cancer screening and diagnosis [40–43]. The case of breast cancer can be minimized through early screening and diagnosis, but the fear of breast cancer plays a vital role in doing that. Indeed, prior research has demonstrated that presenting false or alarming information about breast cancer might make young women wonder if they should also have screenings regularly, which frequently calls into question the advice for mammography screening [44].

This study has several limitations that should be noted. The BCFS's one-factor solution was first verified using the same sample that was split into two groups; hence, additional crossvalidation using new samples is necessary to verify the factor structure. Second, more research is required to evaluate the scale's predictive validity over time, as the study only looked at the factorial validity, concurrent construct validity, and stability of the scale [23]. Third, the results' ability to be broadly applied is restricted by the use of an online convenience sample. The imbalance in the sample, which is dominated by women, further limits how broadly the findings may be applied. More representative community samples must be used for replication to increase the relevance of these findings. Fourth, research on mediating hypotheses including psychological variables connected to breast cancer fear should be conducted in the future. Finally, while the scale showed significant concurrent validity and reliability, its predictive validity is still unknown. Future studies should look into whether the scale can accurately predict real health behaviors, such as the chance of getting screened for breast cancer or practicing preventative care.

## 5. Conclusion

In conclusion, the psychometric validation of the Bangla version of the BCFS demonstrates that it is a reliable and valid tool for assessing breast cancer fear among female university students in Bangladesh. Despite being the very first study on this topic in Bangladesh, we reveal that the BCFS has significant psychometric qualities and is a useful tool for evaluating Bangladeshi women's fear of breast cancer. The findings of this study should be considered when validating the BCFS, particularly in Bangladesh where breast cancer is the most prevalent cancer among women. The outcomes of the BCFS are congruent with the one-factor structure obtained by Champion et al. [12] in their study of the development of the CBCFS. These findings imply that the scale is a legitimate and acceptable method for estimating Bangladeshi women's fear of breast cancer. Nonetheless, to guarantee the scale's wide application, slight differences in item performance among subsamples call for more research. To ensure the scale's efficacy across a range of subgroups, future research ought to investigate these variations and take them into account.

## Figures and Tables

**Table 1:** Background characteristics of the study participants.

	**Total sample (** **N** = 456**)**	**Split Sample 1 (** **N** = 224**)**	**Split Sample 2 (** **N** = 232**)**
	**Number (%)**	**Number (%)**	**Number (%)**
Age of the respondents	Mean: 22.91 (SD 1.12)	Mean: 23.01 (SD 1.53)	Mean: 22.80 (SD 1.71)
Marital status			
Unmarried	378 (82.9)	190 (81.9)	188 (83.9)
Married	78 (17.1)	42 (18.1)	36 (16.1)
Monthly family income level			
< 10,000	53 (11.6)	24 (10.3)	29 (12.9)
10,000–30,000	184 (40.4)	91 (39.2)	93 (41.5)
30,001–50,000	131 (28.7)	66 (28.4)	65 (29.0)
> 50,001	88 (19.3)	51 (22.0)	37 (16.5)
Smoking history			
No	430 (94.3)	224 (96.6)	206 (92.0)
Yes	26 (5.7)	8 (3.4)	18 (8.0)
Place of birth			
Urban	275 (60.3)	149 (64.2)	126 (56.3)
Rural	181 (39.7)	83 (35.8)	98 (43.8)

**Table 2:** Factor loadings in exploratory and confirmatory factor analysis and Cronbach's alpha for Breast Cancer Fear Scale for the overall and the two split samples.

	**Mean (SD)**	**Total sample (** **N** = 456**)**		**Split Sample 1 (** **N** = 224**)**		**Split Sample 2 (** **N** = 232**)**	
	**Total**	**EFA**	**CFA**	**EFA**	**CFA**	**EFA**	**CFA**
Fear1	4.09 (0.88)	0.733	0.679	0.602	0.539	0.832	0.795
Fear2	3.98 (0.90)	0.860	0.827	0.834	0.769	0.879	0.876
Fear3	4.03 (0.90)	0.893	0.905	0.886	0.904	0.899	0.908
Fear4	3.91 (0.95)	0.904	0.920	0.899	0.921	0.908	0.914
Fear5	3.83 (0.99)	0.851	0.818	0.870	0.847	0.835	0.797
Fear6	3.59 (0.99)	0.821	0.755	0.813	0.738	0.834	0.775
Fear7	3.88 (0.90)	0.797	0.722	0.827	0.742	0.772	0.711
Fear8	3.83 (0.96)	0.846	0.792	0.852	0.796	0.843	0.788
KMO		0.934		0.918		0.937	
Cronbach's alpha		0.939		0.933		0.945	
McDonald's		0.940		0.934		0.946	

*Note:* Fear 1: The thought of breast cancer scares me; Fear 2: When I think about breast cancer, I feel nervous; Fear 3: When I think about breast cancer, I get upset; Fear 4: When I think about breast cancer, I get depressed; Fear 5: When I think about breast cancer, I get jittery; Fear 6: When I think about breast cancer, my heart beats faster; Fear 7: When I think about breast cancer, I feel uneasy; Fear 8: When I think about breast cancer, I feel anxious.

**Table 3:** Model fit indices for confirmatory factor analysis across different samples.

**Test indices**	**Total sample (** **N** = 456**)**		**Split Sample 1 (** **N** = 232**)**	**Split Sample 2 (** **N** = 224**)**
	**Test standard**	**Obtained results**	**Obtained results**	**Obtained results**
RMSEA	≤ 0.08	0.071	0.064	0.075
GFI	≥ 0.9	0.974	0.969	0.966
AGFI	≥ 0.9	0.938	0.925	0.918
NFI	≥ 0.9	0.983	0.980	0.978
TLI	≥ 0.9	0.978	0.982	0.977
CFI	≥ 0.9	0.988	0.990	0.988
PGFI	≥ 0.5	0.406	0.404	0.402
SRMR	≤ 0.05	0.022	0.026	0.022
Composite reliability	≤ 0.7	0.918	0.905	0.933
AVE	≥ 0.5	0.650	0.624	0.678

## Data Availability

Data are available from the corresponding author upon reasonable request.

## Supporting Information

Supporting Information Additional supporting information can be found online in the Supporting Information section. Associated supporting files contain inter-item correlation and the results of MICOM measurement invariance and MGA multigroup analysis.
