# Supplementary material for: Psychometric Validation of the Bangla Version of the Breast Cancer Fear Scale Among Female University Students in Bangladesh
Source: Int J Breast Cancer. 2025 Apr 22;2025:6811105. doi: 10.1155/ijbc/6811105 (PMC12041632; doi:10.1155/ijbc/6811105)
Supplement: Supporting Information — Additional supporting information can be found online in the Supporting Information section. Associated supporting files contain inter-item correlation and the results of MICOM measurement invariance and MGA multigroup analysis. [file 6811105.f1.docx]

Table 1. Overall inter-item correlation

| **Correlations** | | | | | | | | | | |
| --- | --- | --- | --- | --- | --- | --- | --- | --- | --- | --- |
|  | | **Fear1** | **Fear2** | **Fear3** | **Fear4** | **Fear5** | **Fear6** | **Fear7** | **Fear8** | **Score Total** |
| **Fear1** | Pearson Correlation | 1 | .675^**^ | .646^**^ | .610^**^ | .544^**^ | .488^**^ | .482^**^ | .529^**^ | .736^**^ |
|  | Sig. (2-tailed) |  | .000 | .000 | .000 | .000 | .000 | .000 | .000 | .000 |
|  | N | 456 | 456 | 456 | 456 | 456 | 456 | 456 | 456 | 456 |
| **Fear2** | Pearson Correlation | .675^**^ | 1 | .768^**^ | .753^**^ | .648^**^ | .631^**^ | .630^**^ | .657^**^ | .856^**^ |
|  | Sig. (2-tailed) | .000 |  | .000 | .000 | .000 | .000 | .000 | .000 | .000 |
|  | N | 456 | 456 | 456 | 456 | 456 | 456 | 456 | 456 | 456 |
| **Ear3** | Pearson Correlation | .646^**^ | .768^**^ | 1 | .838^**^ | .731^**^ | .644^**^ | .639^**^ | .697^**^ | .888^**^ |
|  | Sig. (2-tailed) | .000 | .000 |  | .000 | .000 | .000 | .000 | .000 | .000 |
|  | N | 456 | 456 | 456 | 456 | 456 | 456 | 456 | 456 | 456 |
| **Fear4** | Pearson Correlation | .610^**^ | .753^**^ | .838^**^ | 1 | .760^**^ | .708^**^ | .641^**^ | .725^**^ | .901^**^ |
|  | Sig. (2-tailed) | .000 | .000 | .000 |  | .000 | .000 | .000 | .000 | .000 |
|  | N | 456 | 456 | 456 | 456 | 456 | 456 | 456 | 456 | 456 |
| **Fear5** | Pearson Correlation | .544^**^ | .648^**^ | .731^**^ | .760^**^ | 1 | .704^**^ | .642^**^ | .662^**^ | .853^**^ |
|  | Sig. (2-tailed) | .000 | .000 | .000 | .000 |  | .000 | .000 | .000 | .000 |
|  | N | 456 | 456 | 456 | 456 | 456 | 456 | 456 | 456 | 456 |
| **Fear6** | Pearson Correlation | .488^**^ | .631^**^ | .644^**^ | .708^**^ | .704^**^ | 1 | .629^**^ | .703^**^ | .826^**^ |
|  | Sig. (2-tailed) | .000 | .000 | .000 | .000 | .000 |  | .000 | .000 | .000 |
|  | N | 456 | 456 | 456 | 456 | 456 | 456 | 456 | 456 | 456 |
| **Fear7** | Pearson Correlation | .482^**^ | .630^**^ | .639^**^ | .641^**^ | .642^**^ | .629^**^ | 1 | .696^**^ | .798^**^ |
|  | Sig. (2-tailed) | .000 | .000 | .000 | .000 | .000 | .000 |  | .000 | .000 |
|  | N | 456 | 456 | 456 | 456 | 456 | 456 | 456 | 456 | 456 |
| **Fear8** | Pearson Correlation | .529^**^ | .657^**^ | .697^**^ | .725^**^ | .662^**^ | .703^**^ | .696^**^ | 1 | .847^**^ |
|  | Sig. (2-tailed) | .000 | .000 | .000 | .000 | .000 | .000 | .000 |  | .000 |
|  | N | 456 | 456 | 456 | 456 | 456 | 456 | 456 | 456 | 456 |
| **Score Total** | Pearson Correlation | .736^**^ | .856^**^ | .888^**^ | .901^**^ | .853^**^ | .826^**^ | .798^**^ | .847^**^ | 1 |
|  | Sig. (2-tailed) | .000 | .000 | .000 | .000 | .000 | .000 | .000 | .000 |  |
|  | N | 456 | 456 | 456 | 456 | 456 | 456 | 456 | 456 | 456 |
| **. Correlation is significant at the 0.01 level (2-tailed). | | | | | | | | | | |

Table 2. Split first half inter-item correlation

| **Correlations** | | | | | | | | | | |
| --- | --- | --- | --- | --- | --- | --- | --- | --- | --- | --- |
|  | | **Fear1** | **Fear2** | **Fear3** | **Fear4** | **Fear5** | **Fear6** | **Fear7** | **Fear8** | **Score Total** |
| **Fear1** | Pearson Correlation | 1 | .571^**^ | .528^**^ | .501^**^ | .408^**^ | .363^**^ | .377^**^ | .399^**^ | .618^**^ |
|  | Sig. (2-tailed) |  | .000 | .000 | .000 | .000 | .000 | .000 | .000 | .000 |
|  | N | 232 | 232 | 232 | 232 | 232 | 232 | 232 | 232 | 232 |
| **Fear2** | Pearson Correlation | .571^**^ | 1 | .710^**^ | .701^**^ | .622^**^ | .589^**^ | .692^**^ | .636^**^ | .833^**^ |
|  | Sig. (2-tailed) | .000 |  | .000 | .000 | .000 | .000 | .000 | .000 | .000 |
|  | N | 232 | 232 | 232 | 232 | 232 | 232 | 232 | 232 | 232 |
| **Ear3** | Pearson Correlation | .528^**^ | .710^**^ | 1 | .841^**^ | .759^**^ | .627^**^ | .646^**^ | .698^**^ | .880^**^ |
|  | Sig. (2-tailed) | .000 | .000 |  | .000 | .000 | .000 | .000 | .000 | .000 |
|  | N | 232 | 232 | 232 | 232 | 232 | 232 | 232 | 232 | 232 |
| **Fear4** | Pearson Correlation | .501^**^ | .701^**^ | .841^**^ | 1 | .781^**^ | .683^**^ | .666^**^ | .718^**^ | .895^**^ |
|  | Sig. (2-tailed) | .000 | .000 | .000 |  | .000 | .000 | .000 | .000 | .000 |
|  | N | 232 | 232 | 232 | 232 | 232 | 232 | 232 | 232 | 232 |
| **Fear5** | Pearson Correlation | .408^**^ | .622^**^ | .759^**^ | .781^**^ | 1 | .749^**^ | .663^**^ | .707^**^ | .868^**^ |
|  | Sig. (2-tailed) | .000 | .000 | .000 | .000 |  | .000 | .000 | .000 | .000 |
|  | N | 232 | 232 | 232 | 232 | 232 | 232 | 232 | 232 | 232 |
| **Fear6** | Pearson Correlation | .363^**^ | .589^**^ | .627^**^ | .683^**^ | .749^**^ | 1 | .639^**^ | .684^**^ | .816^**^ |
|  | Sig. (2-tailed) | .000 | .000 | .000 | .000 | .000 |  | .000 | .000 | .000 |
|  | N | 232 | 232 | 232 | 232 | 232 | 232 | 232 | 232 | 232 |
| **Fear7** | Pearson Correlation | .377^**^ | .692^**^ | .646^**^ | .666^**^ | .663^**^ | .639^**^ | 1 | .740^**^ | .825^**^ |
|  | Sig. (2-tailed) | .000 | .000 | .000 | .000 | .000 | .000 |  | .000 | .000 |
|  | N | 232 | 232 | 232 | 232 | 232 | 232 | 232 | 232 | 232 |
| **Fear8** | Pearson Correlation | .399^**^ | .636^**^ | .698^**^ | .718^**^ | .707^**^ | .684^**^ | .740^**^ | 1 | .851^**^ |
|  | Sig. (2-tailed) | .000 | .000 | .000 | .000 | .000 | .000 | .000 |  | .000 |
|  | N | 232 | 232 | 232 | 232 | 232 | 232 | 232 | 232 | 232 |
| **Score Total** | Pearson Correlation | .618^**^ | .833^**^ | .880^**^ | .895^**^ | .868^**^ | .816^**^ | .825^**^ | .851^**^ | 1 |
|  | Sig. (2-tailed) | .000 | .000 | .000 | .000 | .000 | .000 | .000 | .000 |  |
|  | N | 232 | 232 | 232 | 232 | 232 | 232 | 232 | 232 | 232 |
| **. Correlation is significant at the 0.01 level (2-tailed). | | | | | | | | | | |

Table 3. Split half the second inter-item correlation

| **Correlations** | | | | | | | | | | |
| --- | --- | --- | --- | --- | --- | --- | --- | --- | --- | --- |
|  | | **Fear1** | **Fear2** | **Fear3** | **Fear4** | **Fear5** | **Fear6** | **Fear7** | **Fear8** | **Score Total** |
| Fear1 | Pearson Correlation | 1 | .757^**^ | .743^**^ | .701^**^ | .649^**^ | .594^**^ | .577^**^ | .636^**^ | .830^**^ |
|  | Sig. (2-tailed) |  | .000 | .000 | .000 | .000 | .000 | .000 | .000 | .000 |
|  | N | 224 | 224 | 224 | 224 | 224 | 224 | 224 | 224 | 224 |
| Fear2 | Pearson Correlation | .757^**^ | 1 | .816^**^ | .798^**^ | .669^**^ | .672^**^ | .574^**^ | .675^**^ | .875^**^ |
|  | Sig. (2-tailed) | .000 |  | .000 | .000 | .000 | .000 | .000 | .000 | .000 |
|  | N | 224 | 224 | 224 | 224 | 224 | 224 | 224 | 224 | 224 |
| Ear3 | Pearson Correlation | .743^**^ | .816^**^ | 1 | .834^**^ | .708^**^ | .666^**^ | .631^**^ | .697^**^ | .894^**^ |
|  | Sig. (2-tailed) | .000 | .000 |  | .000 | .000 | .000 | .000 | .000 | .000 |
|  | N | 224 | 224 | 224 | 224 | 224 | 224 | 224 | 224 | 224 |
| Fear4 | Pearson Correlation | .701^**^ | .798^**^ | .834^**^ | 1 | .743^**^ | .734^**^ | .615^**^ | .731^**^ | .906^**^ |
|  | Sig. (2-tailed) | .000 | .000 | .000 |  | .000 | .000 | .000 | .000 | .000 |
|  | N | 224 | 224 | 224 | 224 | 224 | 224 | 224 | 224 | 224 |
| Fear5 | Pearson Correlation | .649^**^ | .669^**^ | .708^**^ | .743^**^ | 1 | .669^**^ | .624^**^ | .624^**^ | .840^**^ |
|  | Sig. (2-tailed) | .000 | .000 | .000 | .000 |  | .000 | .000 | .000 | .000 |
|  | N | 224 | 224 | 224 | 224 | 224 | 224 | 224 | 224 | 224 |
| Fear6 | Pearson Correlation | .594^**^ | .672^**^ | .666^**^ | .734^**^ | .669^**^ | 1 | .624^**^ | .722^**^ | .838^**^ |
|  | Sig. (2-tailed) | .000 | .000 | .000 | .000 | .000 |  | .000 | .000 | .000 |
|  | N | 224 | 224 | 224 | 224 | 224 | 224 | 224 | 224 | 224 |
| Fear7 | Pearson Correlation | .577^**^ | .574^**^ | .631^**^ | .615^**^ | .624^**^ | .624^**^ | 1 | .655^**^ | .775^**^ |
|  | Sig. (2-tailed) | .000 | .000 | .000 | .000 | .000 | .000 |  | .000 | .000 |
|  | N | 224 | 224 | 224 | 224 | 224 | 224 | 224 | 224 | 224 |
| Fear8 | Pearson Correlation | .636^**^ | .675^**^ | .697^**^ | .731^**^ | .624^**^ | .722^**^ | .655^**^ | 1 | .845^**^ |
|  | Sig. (2-tailed) | .000 | .000 | .000 | .000 | .000 | .000 | .000 |  | .000 |
|  | N | 224 | 224 | 224 | 224 | 224 | 224 | 224 | 224 | 224 |
| Score Total | Pearson Correlation | .830^**^ | .875^**^ | .894^**^ | .906^**^ | .840^**^ | .838^**^ | .775^**^ | .845^**^ | 1 |
|  | Sig. (2-tailed) | .000 | .000 | .000 | .000 | .000 | .000 | .000 | .000 |  |
|  | N | 224 | 224 | 224 | 224 | 224 | 224 | 224 | 224 | 224 |
| **. Correlation is significant at the 0.01 level (2-tailed). | | | | | | | | | | |

Table 4 and forward: Test of the Results of MICOM Measurement invariance and MGA Multigroup Analysis

|  | **Original correlation** | **Correlation permutation mean** | **5.0%** | **Permutation p value** |
| --- | --- | --- | --- | --- |
| **Step 2** | 0.999 | 0.921 | 0.987 | 0.981 |

|  | **Original difference** | **Permutation mean difference** | **2.5%** | **97.5%** | **Permutation p value** |
| --- | --- | --- | --- | --- | --- |
| **Step 3a (men)** | 0.144 | 0.000 | -0.175 | 0.192 | 0.141 |
| **Step 3b (variance)** | -0.135 | -0.008 | -0.329 | 0.323 | 0.410 |

|  | **Original (residence_1)** | **Original (residence_0)** | **Original difference** | **Permutation mean difference** | **2.5%** | **97.5%** | **Permutation p value** |
| --- | --- | --- | --- | --- | --- | --- | --- |
| **Total effects** | -0.279 | -0.250 | -0.029 | 0.033 | -0.563 | 0.571 | 0.775 |

| Outer Loadings | **Original (residence_1)** | **Original (residence_0)** | **Original difference** | **Permutation mean difference** | **2.5%** | **97.5%** | **Permutation p value** |
| --- | --- | --- | --- | --- | --- | --- | --- |
| **Fear1 <- Fear** | 0.680 | 0.771 | -0.091 | -0.004 | -0.187 | 0.160 | 0.213 |
| **Fear2 <- Fear** | 0.854 | 0.868 | -0.014 | -0.008 | -0.121 | 0.084 | 0.753 |
| **Fear3 <- Fear** | 0.909 | 0.877 | 0.032 | -0.007 | -0.096 | 0.076 | 0.377 |
| **Fear4 <- Fear** | 0.902 | 0.887 | 0.015 | -0.009 | -0.138 | 0.094 | 0.767 |
| **Fear5 <- Fear** | 0.857 | 0.846 | 0.012 | -0.005 | -0.132 | 0.118 | 0.839 |
| **Fear6 <- Fear** | 0.805 | 0.824 | -0.019 | -0.008 | -0.132 | 0.100 | 0.647 |
| **Fear7 <- Fear** | 0.794 | 0.798 | -0.004 | -0.012 | -0.189 | 0.130 | 0.951 |
| **Fear8 <- Fear** | 0.788 | 0.895 | -0.107 | -0.010 | -0.136 | 0.106 | 0.056 |

| **Outer weights** | **Original (residence_1)** | **Original (residence_0)** | **Original difference** | **Permutation mean difference** | **2.5%** | **97.5%** | **Permutation p value** |
| --- | --- | --- | --- | --- | --- | --- | --- |
| **Fear1 <- Fear** | 0.146 | 0.127 | 0.019 | 0.000 | -0.137 | 0.144 | 0.775 |
| **Fear2 <- Fear** | 0.175 | 0.167 | 0.008 | -0.005 | -0.131 | 0.129 | 0.859 |
| **Fear3 <- Fear** | 0.175 | 0.147 | 0.028 | -0.002 | -0.099 | 0.082 | 0.532 |
| **Fear4 <- Fear** | 0.132 | 0.096 | 0.036 | 0.002 | -0.185 | 0.156 | 0.729 |
| **Fear5 <- Fear** | 0.155 | 0.164 | -0.009 | 0.002 | -0.100 | 0.121 | 0.844 |
| **Fear6 <- Fear** | 0.135 | 0.135 | 0.001 | 0.002 | -0.126 | 0.103 | 0.990 |
| **Fear7 <- Fear** | 0.122 | 0.141 | -0.019 | -0.003 | -0.135 | 0.093 | 0.716 |
| **Fear8 <- Fear** | 0.171 | 0.201 | -0.030 | -0.005 | -0.128 | 0.129 | 0.566 |

| AVE | **Original (residence_1)** | **Original (residence_0#2)** | **Original difference** | **Permutation mean difference** | **2.5%** | **97.5%** | **Permutation p value** |
| --- | --- | --- | --- | --- | --- | --- | --- |
| **Fear** | 0.683 | 0.717 | -0.034 | -0.002 | -0.086 | 0.073 | 0.431 |

| Composite Reliability | **Original (residence_1)** | **Original (residence_0)** | **Original difference** | **Permutation mean difference** | **2.5%** | **97.5%** | **Permutation p value** |
| --- | --- | --- | --- | --- | --- | --- | --- |
| Composite Reliability (rho_c) | 0.945 | 0.953 | -0.008 | -0.001 | -0.020 | 0.017 | 0.410 |
| Composite Reliability (rho_a) | 0.938 | 0.957 | -0.019 | 0.003 | -0.033 | 0.037 | 0.284 |
| Cronbach Alpha | 0.932 | 0.943 | -0.011 | -0.000 | -0.025 | 0.021 | 0.350 |
